# Supplementary material for: Coastal drowning: A scoping review of burden, risk factors, and prevention strategies
Source: PLoS One. 2021 Feb 1;16(2):e0246034. doi: 10.1371/journal.pone.0246034 (PMC7850505; doi:10.1371/journal.pone.0246034)
Supplement: S2 File — (PDF) [file pone.0246034.s004.pdf]

Supplementary File 2: Coastal Drowning Scoping Review Search Strategies

MEDLINE

1. drown*.ti,ab.
2. Drowning/ep, mo, pc, sn [Epidemiology, Mortality, Prevention & Control, Statistics & Numerical Data]
3. 1 or 2
4. "oceans and seas"/ or bays/ or estuaries/ or Seawater/ or "Bathing Beaches"/
5. (ocean or sea or bay* or estuar* or beach* or lagoon* or harbo?r* or surf or coast*).mp.
6. (site* adj5 drown*).mp. [mp=title, abstract, original title, name of substance word, subject heading word, floating sub-heading word, keyword heading word, organism supplementary concept word, protocol supplementary concept word, rare disease supplementary concept word, unique identifier, synonyms]
7. (location* adj5 drown*).mp. [mp=title, abstract, original title, name of substance word, subject heading word, floating sub-heading word, keyword heading word, organism supplementary concept word, protocol supplementary concept word, rare disease supplementary concept word, unique identifier, synonyms]
8. Swimming Pools/ or pool*.mp.
9. 4 or 5 or 6 or 7 or 8
10. 3 and 9
11. limit 10 to humans
12. limit 11 to (english or spanish)
13. limit 12 to (case reports or clinical conference or comment or editorial or letter)
14. 12 not 13

MEDLINE Epub

1. drown*.mp. [mp=title, abstract, original title, name of substance word, subject heading word, floating sub-heading word, keyword heading word, organism supplementary concept word, protocol supplementary concept word, rare disease supplementary concept word, unique identifier, synonyms]
2. (ocean or sea or bay* or estuar* or beach* or lagoon* or harbo?r* or surf or coast*).mp.
3. (site* adj5 drown*).mp.
4. (location* adj5 drown*).mp.
5. ("swimming pool" or "swimming pools" or pool or pools).mp. [mp=title, abstract, original title, name of substance word, subject heading word, floating sub-heading word, keyword heading word, organism supplementary concept word, protocol supplementary concept word, rare disease supplementary concept word, unique identifier, synonyms]
6. 2 or 3 or 4 or 5
7. 1 and 6

8. limit 7 to humans
9. limit 7 to (case reports or comment or editorial)
10. 7 not 9
11. limit 10 to (english or spanish)

EMBASE

1. drown*.mp.
2. sea/ or ocean environment/ or coastal waters/ or intertidal zone/ or bay/ or estuary/ or seashore/ or sea water/ or lagoon/
3. (ocean or sea or bay* or estuar* or beach* or lagoon* or harbo?r* or surf or coast*).mp.
4. (location* adj5 drown*).mp.
5. (site* adj5 drown*).mp.
6. swimming pool/
7. 2 or 3 or 4 or 5 or 6
8. 1 and 7
9. limit 8 to human
10. limit 9 to (english or spanish)
11. limit 10 to (conference abstract or conference paper or "conference review" or editorial or letter)
12. 10 not 11

Environment Complete

S7	S1 AND S4	Limiters - Publication Type: Academic Journal<br>Expanders - Apply equivalent subjects<br>Narrow by Subject: - safety education<br>Narrow by Subject: - problem solving<br>Narrow by Subject: - medical cooperation<br>Narrow by Subject: - lifesaving equipment<br>Narrow by Subject: - life preservers (safety equipment)<br>Narrow by Subject: - government policy<br>Narrow by Subject: - evaluation research<br>Narrow by Subject: - emergency medicine<br>Narrow by Subject: - education<br>Narrow by Subject: - decision making<br>Narrow by Subject: - cpr (first aid)

		Narrow by Subject: - boats & boating<br>Narrow by Subject: - attitudes toward health<br>Narrow by Subject: - accident prevention<br>Narrow by Subject: - public safety<br>Narrow by Subject: - surfing<br>Narrow by Subject: - rescues<br>Narrow by Subject: - recreation<br>Narrow by Subject: - life jackets (garments)<br>Narrow by Subject: - vulnerability (psychology)<br>Narrow by Subject: - swimmers<br>Narrow by Subject: - risk perception<br>Narrow by Subject: - rescue work<br>Narrow by Subject: - lifesaving<br>Narrow by Subject: - drowning victims<br>Narrow by Subject: - death<br>Narrow by Subject: - aquatic sports safety measures<br>Narrow by Subject: - causes of death<br>Narrow by Subject: - beachgoers<br>Narrow by Subject: - work-related injuries<br>Narrow by Subject: - lifeguards<br>Narrow by Subject: - mortality<br>Narrow by Subject: - swimming<br>Narrow by Subject: - accidents<br>Narrow by Subject: - drownproofing<br>Narrow by Subject: - rip currents<br>Narrow by Subject: - drowning<br>Search modes - Boolean/Phrase	
	S6	S1 AND S4	Limiters - Publication Type: Academic Journal<br>Expanders - Apply equivalent subjects<br>Search modes - Boolean/Phrase
	S5	S1 AND S4	Expanders - Apply equivalent subjects<br>Search modes - Boolean/Phrase
	S4	S2 OR S3	Expanders - Apply equivalent subjects<br>Search modes - Boolean/Phrase

	S3	(site* N5 drown*) OR (location* N5 drown*)	Expanders - Apply equivalent subjects Search modes - Boolean/Phrase
	S2	bay* OR beach* OR coast* OR estuary OR harbor* OR harbour* OR lagoon* OR ocean OR sea* OR surf	Expanders - Apply equivalent subjects Search modes - Boolean/Phrase
	S1	drown*	Expanders - Apply equivalent subjects Search modes - Boolean/Phrase

CINAHL

S6	S1 AND S4	Limiters - Human; Publication Type: Journal Article, Meta Analysis, Randomized Controlled Trial, Research, Review, Statistics, Systematic Review; Language: English, Spanish
		Expanders - Apply equivalent subjects
		Narrow by SubjectMajor: - equipment design
		Narrow by SubjectMajor: - environment<br>Narrow by SubjectMajor: - emergency service<br>Narrow by SubjectMajor: - decision making<br>Narrow by SubjectMajor: - community programs<br>Narrow by SubjectMajor: - athletic injuries<br>Narrow by SubjectMajor: - respiration, artificial<br>Narrow by SubjectMajor: - emergency medical services<br>Narrow by SubjectMajor: - death<br>Narrow by SubjectMajor: - child safety

		Narrow by SubjectMajor: - child abuse<br><br>Narrow by SubjectMajor: - accidental falls<br><br>Narrow by SubjectMajor: - risk taking behavior<br><br>Narrow by SubjectMajor: - aquatic sports<br><br>Narrow by SubjectMajor: - accidents<br><br>Narrow by SubjectMajor: - risk assessment<br><br>Narrow by SubjectMajor: - bathing and baths<br><br>Narrow by SubjectMajor: - water<br><br>Narrow by SubjectMajor: - ships<br><br>Narrow by SubjectMajor: - oceans and seas<br><br>Narrow by SubjectMajor: - natural environment<br><br>Narrow by SubjectMajor: - near drowning<br><br>Narrow by SubjectMajor: - accidents, occupational<br><br>Narrow by SubjectMajor: - rescue work<br><br>Narrow by SubjectMajor: - safety<br><br>Narrow by SubjectMajor: - resuscitation, cardiopulmonary<br><br>Narrow by SubjectMajor: - cause of death<br><br>Narrow by SubjectMajor: - wounds and injuries<br><br>Narrow by SubjectMajor: - swimming<br><br>Narrow by SubjectMajor: - drowning<br><br>Search modes - Boolean/Phrase	
	S5	S1 AND S4	Limiters - Human; Publication Type: Journal Article, Meta Analysis, Randomized Controlled

			Trial, Research, Review, Statistics, Systematic Review; Language: English, Spanish<br><br>Expanders - Apply equivalent subjects<br><br>Search modes - Boolean/Phrase
	S4	S2 OR S3	Expanders - Apply equivalent subjects<br><br>Search modes - Boolean/Phrase
	S3	(site* N5 drown*) OR (location* N5 drown*)	Expanders - Apply equivalent subjects<br><br>Search modes - Boolean/Phrase
	S2	bay* OR beach* OR coast* OR estuary OR harbor* OR harbour* OR lagoon* OR ocean OR sea* OR surf OR pool	Expanders - Apply equivalent subjects<br><br>Search modes - Boolean/Phrase
	S1	drown*	Expanders - Apply equivalent subjects<br><br>Search modes - Boolean/Phrase

SportDiscus

S5	S1 AND S4	Limiters - Language: English, Spanish; Publication Type: Academic Journal<br><br>Expanders - Apply equivalent subjects<br><br>Search modes - Boolean/Phrase	
	S4	S2 OR S3	Expanders - Apply equivalent subjects<br><br>Search modes - Boolean/Phrase
	S3	(site* N5 drown*) OR (location* N5 drown*)	Expanders - Apply equivalent subjects<br><br>Search modes - Boolean/Phrase

	S2	bay* OR beach* OR coast* OR estuary OR harbor* OR harbour* OR lagoon* OR ocean OR sea* OR surf	Expanders - Apply equivalent subjects<br><br>Search modes - Boolean/Phrase
	S1	drown*	Expanders - Apply equivalent subjects<br><br>Search modes - Boolean/Phrase
