# Supplementary material for: Coastal drowning: A scoping review of burden, risk factors, and prevention strategies
Source: PLoS One. 2021 Feb 1;16(2):e0246034. doi: 10.1371/journal.pone.0246034 (PMC7850505; doi:10.1371/journal.pone.0246034)
Supplement: S3 File — (PDF) [file pone.0246034.s005.pdf]

Supplementary File 3: Coastal Drowning Scoping Review Data Extraction Form

Study Info

1. First Author Last Name

____

2. Year Published

____

3. Full title of paper

____

4. Country of Study

____

5. Language of Publication

   Mark only one oval.

   ( ) English

   ( ) Spanish

6. Study Period - First Year

____

7. Study Period - Last Year

8. Aim of the study

Burden

9. This paper includes data on:

Mark only one oval.

- All coastal drowning only
- Specific type of coastal drowning only (rips, diving etc)
- Specific type of drowning from any water site (cold water, bystander rescue etc) Drowning only -
- reported multiple bodies of water
- Drowning AND other aquatic fatalities / injuries Many
- different injury topics
- Other: ____________________

10. What outcomes were reported in the study?

Mark only one oval.

- ( ) fatal only
- ( ) fatal and non-fatal
- ( ) "Good" and "Bad" outcome including neurological sequelae Other:
- ( ) ____________________

11. Age groups studied

Mark only one oval.

- ( ) Pediatric only
- ( ) Adults only
- ( ) All ages

12. Total population based or specific population
Mark only one oval.

- ( ) Total population
- ( ) Other: ____________________

13. Geographic catchment area

Describe "Victoria, Aus" or "Los Angeles County, USA"

____
____
____
____
____

14. Intentionality

Mark only one oval.

- ( ) Study only included unintentional cases
- ( ) Study included intentional cases, but differentiated the reported numbers by water site Study discusses or
- ( ) included intentional cases, but did not differentiate in reported coastal numbers
- ( ) Study did NOT address or discuss cases by intentionality in any way Other: _
- ( ) ______________________________

15. Boating

Mark only one oval.

- ( ) Study only included non-boating cases
- ( ) Study included boating cases, but differentiated the reported numbers by water site
- ( ) Study discusses or included boating cases, but did not differentiate in reported coastal number
- ( ) Study did NOT address or discuss boating origin in any way
- ( ) Other: ______________________________

16. Boat / intentional info

Add info here to clarify boating / intentional information.

____

____

____

____

____

17. Data sources for the study

Check all that apply

Check all that apply.

- [ ] Hospital data
- [ ] Death certificates/ coroner data
- [ ] EMS data or registry
- [ ] Beach Lifeguard or Rescue data
- [ ] Other Rescue data (Fire Department, rescue swimmer, coast guard etc.)
- [ ] Newspaper / media
- [ ] Questionnaires Other:
  - [ ] ______________________________

18. Which cases were included in the study?

Check all that apply

Check all that apply.

- [ ] People autopsied by medical examiner
- [ ] People in ICU (includes pediatric ICU)
- [ ] People admitted to hospital
- [ ] People arriving at ED
- [ ] People transported by or receiving care from EMS / ambulance
- [ ] Patients rescued from the water
- [ ] People appearing in newspaper/media reports
- [ ] Other: ______________________________

19. Analysis used

Check all that apply.

- [ ] Descriptive only (no statistical evaluation)
- [ ] Univariate or bivariate
- [ ] Multivariable - confounders identified a priori
- [ ] Multivariable - no specific question /
- [ ] Other: ______________________

20. Did statistical analysis include only coastal drowning?

Mark only one oval.

- ( ) NA- descriptive only
- ( ) Included only coastal drowning cases
- ( ) Included multiple bodies of water
- ( ) Combo- some tests include multiple bodies, some coastal only Other:
- ( ) ______________________

Coastal drowning terminology and numbers

21. Coastal / coast

____

22. Ocean

____

23. Beach

24. Surf

25. Harbour

26. Bay

27. Lagoon

28. Salt water

29. Sea

30. Other

Write word and number with a dash e.g. "Marsh - 22" (if multiple separate with semi-colon)

31. Issues with reporting sub-categories?

leave blank unless need to describe any issues reporting sub-categories.

Other numbers info

32. Total number of fatal drowning cases reported in the study

33. Percentage of reported coastal fatalities from multisite studies

34. Rank of proportion in multisite studies

35. Reported coastal drowning rate info from paper

36. Activity prior to coastal drowning

37. Any other clarifying notes?

Risk Factors

38. Is this risk factor specific to COASTAL drowning?

Only include if the risk factor is specific to coastal drowning. Do NOT include if coastal drowning was included with other water sites to come up with a different risk factor.

Mark only one oval.

- ( ) Yes
- ( ) No

39. What is the risk factor: be specific.

40. What is the risk factor outcome?

41. How was the risk factor identified

Mark only one oval.

- ⬭ Reported higher population-based prevalence / incidence rate
- ⬭ Reported higher percentage or number among cases
- ⬭ Statistically significant univariate/bivariate correlation among cases
- ⬭ Statistically significant multivariable correlation
- ⬭ Information obtained from a survey or questionnaire reported in paper without supporting evidence/data
- ⬭ Other: ____________________

42. Identification notes

Notes to explain how this was included as a risk factor

____
____
____
____
____

43. Add another risk factor?

Mark only one oval.

- ⬭ Yes - add a different risk factor (Repeat)
- ⬭ No - I have added all the risk factors, move on.

Prevention Strategies

44. Did the study include prevention strategies?

Mark only one oval.

- ⬭ Yes
- ⬭ No

45. What is the prevention strategy? (Be Specific)

46. For notes only – what does the text from the article say?

47. What is the support/evidence for this prevention strategy?

48. Any other clarifying info?

49. Add another prevention strategy?

Mark only one oval.

- ⬭ Yes - add a different prevention strategy (Repeat)
- ⬭ No - I have added all the prevention strategies, finish.
