# Supplementary material for: Coastal drowning: A scoping review of burden, risk factors, and prevention strategies
Source: PLoS One. 2021 Feb 1;16(2):e0246034. doi: 10.1371/journal.pone.0246034 (PMC7850505; doi:10.1371/journal.pone.0246034)
Supplement: Attachment — Submitted filename: Reviewer comment.docx [file pone.0246034.s006.docx]

Line 264-267:

Could you specify high income, upper-middle income, lower-middle income, and low-income countries?

Line 273-275:

The summation of 62 studies, 32, and 18 studies is not equal to 146.
